# Correlation of TSHR and CTLA-4 Single Nucleotide Polymorphisms with Graves Disease

**DOI:** 10.1155/2019/6982623

**Published:** 2019-09-03

**Authors:** Weihua Sun, Xiaomei Zhang, Jing Wu, Wendi Zhao, Shuangxia Zhao, Minglong Li

**Affiliations:** ^1^Department of Endocrinology, Shandong Provincial Hospital Affiliated to Shandong University, Jinan, 250000 Shandong Province, China; ^2^Department of Endocrinology, The First Affiliated Hospital of Bengbu Medical College, Bengbu, 233000 Anhui Province, China; ^3^The Core Laboratory in Medical Center of Clinical Research, Department of Endocrinology, Shanghai Ninth People's Hospital, Shanghai Jiaotong University (SJTU) School of Medicine, Shanghai 200011, China

## Abstract

This study was designed to explore the association between Graves disease (GD) and thyroid-stimulating hormone receptor (TSHR) and cytotoxic T-lymphocyte-associated antigen 4 (CTLA-4) single nucleotide polymorphisms (SNPs). We studied a total of 1217 subjects from a Han population in northern Anhui province in China. Six SNPs within TSHR (rs179247, rs12101261, rs2284722, rs4903964, rs2300525, and rs17111394) and four SNPs within CTLA-4 (rs10197319, rs231726, rs231804, and rs1024161) were genotyped via a Taqman probe technique using a Fluidigm EP1 platform. The TSHR alleles rs179247-G, rs12101261-C, and rs4903964-G were negatively correlated with GD, whereas the rs2284722-A and rs17111394-C alleles were positively correlated with GD. Analyzing TSHR SNPs at rs179247, rs2284722, rs12101261, and rs4903964 yielded 8 different haplotypes. There were positive correlations between GD risk and the haplotypes AGTA and AATA (OR = 1.27, 95%CI = 1.07‐1.50, *P* = 0.005; OR = 1.45, 95%CI = 1.21‐1.75, *P* < 0.001, respectively). There were negative correlations between GD risk and the haplotype GGCG (OR = 0.56, 95%CI = 0.46‐0.67, *P* < 0.001). With respect to haplotypes based on SNPs at the TSHR rs2300525 and rs17111394 loci, the CC haplotype was positively correlated with GD risk (OR = 1.32, 95%CI = 1.08‐1.60, *P* = 0.006). Analyzing CTLA-4 SNPs at rs231804, rs1024161, and rs231726 yielded four haplotypes, of which AAA was positively correlated with GD risk (OR = 1.21, 95%CI = 1.02‐1.43, *P* = 0.029). Polymorphisms at rs179247, rs12101261, rs2284722, rs4903964, and rs17111394 were associated with GD susceptibility. Haplotypes of both TSHR and CTLA-4 were additionally related to GD risk.

## 1. Introduction

GD is a common organ-specific autoimmune disease and the most common cause of thyrotoxicosis. At present, however, the molecular mechanisms underlying GD have not been elucidated. GD susceptibility stems from a confluence of genetic, environmental, and immunological factors [1]. In an individual with a genetic predisposition for GD, the disease may develop as a consequence of environmental influences that induce or exacerbate immune dysfunction, ultimately leading to the onset of autoimmunity, with clear evidence for the existence of genetic factors predisposing individuals to autoimmune thyroid disease [2, 3]. A recent study from the National Health and Nutritional Examination Survey further sought to identify the role of genetic susceptibility in the etiology of GD [4]. GD is a complex polygenetic disease, with multiple risk genes influencing its onset. GWAS studies have been very popular in the identification of such susceptibility loci for thyroid autoimmune diseases [5–13].

TSHR is a primary candidate gene believed to be related to GD susceptibility. TSHR is a specific protein expressed in thyroid cells in the thyroid follicular membrane. TSH regulates both thyroid growth and functionality via TSHR signaling. A study by Zhan et al. [14] identified a novel susceptibility loci for serum TSH levels in Chinese populations using a GWAS approach.

TSHR is a member of the G-protein-coupled receptor superfamily encoded on chromosome 14. The protein is a single 764 amino acid peptide chain encoded for across 10 exons with a molecular weight of 84000 daltons. TSH binding to TSHR promotes G-protein signaling, leading to activation of the cAMP and/or phosphoinositide Ca^2+^ signal transduction pathways. There are many antithyroid autoantibodies present in the serum of patients with GD, including thyrotrophin receptor antibody (TRAb), thyroglobulin antibody (TGAb), and thyroid peroxidase antibody (TPOAb). TRAb is an antibody which is specific to TSHR, and it is believed to be the autoantibody most important for the development of hyperthyroidism. Most patients with GD exhibit TRAb autoantibodies in the peripheral blood. TRAb is an immunoglobulin (IgG) that, when present in the serum, competes with TSH to bind to TSHR, activating the receptor and inducing biological effects similar to those of TSH. Research has shown that a targeted immunotherapy strategy in mice aimed at disrupting antigen processing and presentation in HLA-DR3 transgenic mice blocks the immune response to TSHR, thus offering a potential avenue for GD treatment [15]. These findings emphasize the importance of immunological factors in driving GD and highlight the potential value of immunotherapy in its treatment. Further work has confirmed that SNPs in the TSHR gene are associated with defects in central immune tolerance that can lead to the onset of autoimmunity [16].

CTLA-4 is a member of the immunoglobulin gene superfamily and a negative regulator of T cell responses that is associated with immune tolerance. CTLA-4 is expressed on the surface of T cells mainly in the form of a dimer, and when it interacts with its cognate ligands, this induces inhibitory signals which terminate T cell activation and proliferation. Polymorphisms in CTLA-4 may alter its functionality such that the activation of T cells cannot be inhibited, resulting in a loss of immune tolerance and the occurrence of autoimmunity, making it vital that normal CTLA-4 activity be maintained. CTLA-4 is a major susceptibility gene associated with autoimmune thyroid disease (AITD). An association study aimed at identifying SNPs in the CTLA-4 gene present in GD patients and control subjects has confirmed that CTLA-4 is indeed a susceptibility gene for GD in the Chinese Han population [17], and studies in children have confirmed this result [18]. Interactions among SNPs at rs231775, rs231779, and rs3087243 significantly increase an individual's susceptibility to GD [19].

A number of GD susceptibility genes have been identified to date including human leukocyte antigen (HLA) I and II, cluster of differentiation 40 (CD40), TSHR, protein tyrosine phosphatase nonreceptor 22 (PTPN22), interferon-inducible helicase domain 1 (IFIH1), CTLA-4, forkhead Box P3 (FoxP3), Ikaros family of zinc finger3 (IKZF3), FC-receptor-like 3 (FCRL3), and thyroglobulin (TG) [20–27]. Of these, TSHR is a thyroid-specific gene and CTLA-4 is an immunoregulatory gene affecting GD development, both of which are related to the pathogenesis of hyperthyroidism. Individual patient disease phenotypes may be derived from interactions between genetic and environmental factors. Therefore, we carried out a study to assess the relationship between TSHR and CTLA-4 SNPs and GD, and we analyzed the interaction between these two genes in this context. Most previous studies have focused on the TSHR coding region; however, in recent years the relationship between the TSHR noncoding region and GD has been increasingly studied. SNPs in noncoding promoter regions can alter gene expression or localization. Similarly, certain introns contain regulatory elements or regulate transcript splicing, thereby allowing intronic SNPs to control gene expression and function. Chu et al. [5] found the susceptible sites of GD through GWAS screening. Referring to the research results and retrieving the database NCBI (https://www.ncbi.nlm.nih.gov/snp/), we therefore focused on six SNPs in intron 1 of the TSHR gene (rs179247, rs12101261, rs2284722, rs4903964, rs2300525, and rs17111394) and four SNPs in the CTLA-4 gene (rs10197319, rs231726, rs231804, and rs1024161) in order to carry out a case-control study in a Han population from northern Anhui province in China.

## 2. Materials and Methods

We studied a total of 1217 subjects, divided into a case group and a control group. The control group was composed of 620 healthy subjects including 113 males and 507 females. The GD case group was composed of 597 individuals including 127 male patients and 470 female patients. All patients and control subjects were from a Chinese Han population from northern Anhui province and were unrelated to each other. Patients with other autoimmune diseases or a family history thereof were excluded.

Subjects were diagnosed with GD based upon clinical and laboratory examinations that confirmed hyperthyroidism, which was accompanied by symptoms of a high metabolism, diffuse goiter, thyroid ophthalmopathy, and pretibial myxedema, as well as high serum levels of free thyroxine (FT4) and free T3 (FT3), very low levels of circulating thyroid-stimulating hormone (TSH), and positive TRAb circulation. This study was approved by our local ethics committee.

### 2.1. Genotyping of SNPs

From each individual, a 5 mL sample of peripheral blood was collected in an EDTA-treated tube and use for DNA extraction with a DNA purification kit (Fujifilm Company) based on the provided instructions. All DNA samples were genotyped using Illumina Human660-Quad BeadChips. Illumina BeadStudio 3.3 software was used for genotype clustering. All samples had a mean call rate of 99.8%. The genotypes of the TSHR and CTLA-4 gene SNPs were determined using a Taqman probe technique with a Fluidigm EP1 platform. Polymerase chain reaction (PCR) was employed to amplify each target gene sequence as previously described [5]. Our targets were six SNPs in intron 1 of TSHR (rs179247, rs12101261, rs2284722, rs4903964, rs2300525, and rs17111394) and four in CTLA-4 (rs10197319, rs231726, rs231804, and rs1024161).

### 2.2. Statistical Analyses

Quantitative data are given as means ± standard deviations, and differences between groups were compared via *t*-test. Qualitative data were described as percentages or proportions, and differences between groups were compared via the *χ*^2^ test. The genotype and allele frequencies for TSHR and CTLA-4 gene in cases and controls were assessed by chi-square test. The correlation between SNPs and GD was analyzed via a binary classification single-factor and multifactor logistic regression model, and the OR and 95% CI were calculated to assess the relationship between the genotype and incidence of GD. Rank sum test was used for rank data. SPSS v19.0 was used for all above statistical analyses. The Haploview 4.2 software was used to determine whether the distributions of the 10 assessed loci conformed to a Hardy-Weinberg equilibrium in the control group and analyze the relationship between the haplotype of TSHR and CTLA-4 and GD. Dimensionality reduction (MDR) was used to analyze the relationship between gene-gene interactions and the incidence of GD. All the tests were two-tailed, with a test level of *α* = 0.05 and with *P* < 0.05 as the threshold of significance.

## 3. Results

Clinical data is shown in Table 1. We used 1.5 U/L as a cutoff value for TRAb levels. A Hardy-Weinberg equilibrium test was performed on the control group, revealing all 10 SNPS in TSHR and CTLA-4 to conform to a Hardy-Weinberg equilibrium (*P* > 0.05) (Table 2).

In the TSHR gene, there was no difference in the distribution of alleles or genotypes at rs2300525 between the case group and the control group (*P* > 0.05). For rs179247, rs2284722, rs12101261, rs4903964, and rs17111394, the distribution of the corresponding alleles or genotypes between the two groups was statistically significant (*P* < 0.05).

In the CTLA-4 gene, there was no difference in the distribution of alleles or genotypes at sites rs231804, rs1024161, or rs10197319 between the case group and the control group (*P* > 0.05). There was no significant difference in the distribution of the genotype at rs231726 between the two groups (*P* > 0.05), while the distribution difference of alleles at this site between the two groups was statistically significant (*P* < 0.05).

After adjusting for age, a correlation analysis of the association between these ten SNPs and GD revealed that rs179247-G, rs12101261-C, and rs4903964-G were negatively correlated with the incidence of GD in both the male and female populations. In addition, rs2284722-A and rs17111394-C were positively correlated with the incidence of GD in the overall and female populations, while rs2300525-C was positively correlated with the incidence of GD only in the female population. CTLA-4 rs231726-G was negatively correlated with the risk of GD in the overall population. In the dominant model of rs10197319, TT+CT carriers had a higher risk of GD in the overall and male populations than those with the homozygous CC genotype (Table 3).

**Figure 1:**
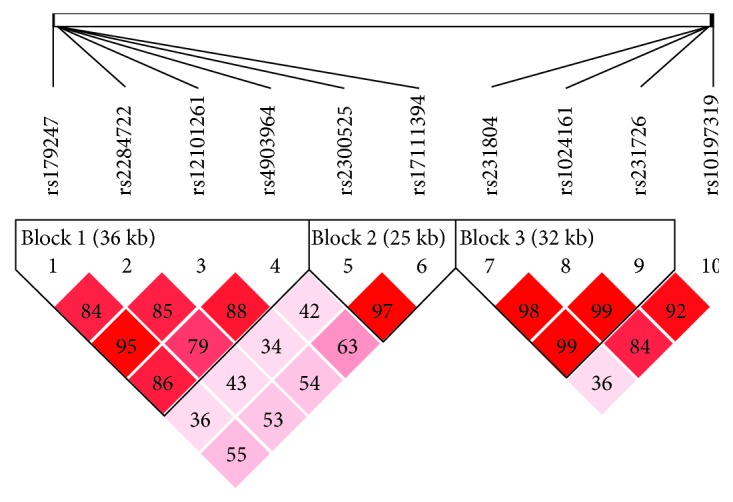
TSHR and CTLA-4 linkage disequilibrium analysis diagram (∣D′∣). TSHR SNP sites rs179247, rs2284722, rs12101261, and rs4903964; TSHR SNP sites rs2300525 and rs17111394; and CTLA-4 sites rs231804, rs1024161, and rs231726 were located in the same haplotype region respectively; ∣D′∣ is the linkage disequilibrium coefficient.

**Figure 2:**
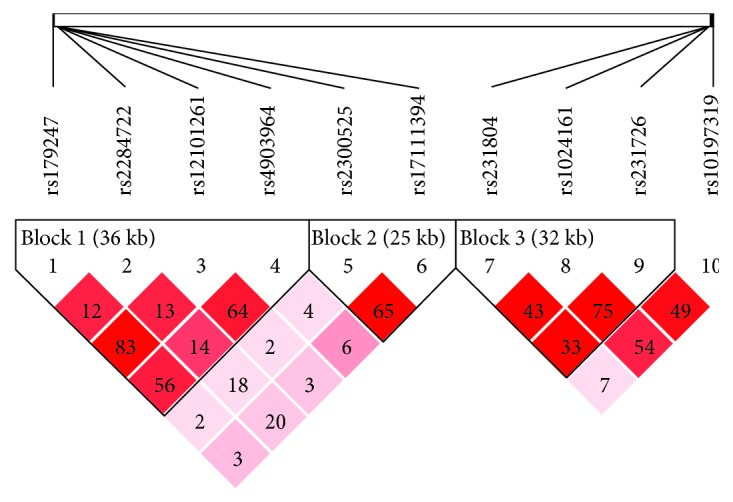
TSHR and CTLA-4 linkage disequilibrium analysis diagram (*r*^2^). TSHR SNP sites rs179247, rs2284722, rs12101261, and rs4903964; TSHR SNP sites rs2300525 and rs17111394; and CTLA-4 sites rs231804, rs1024161, and rs231726 were located in the same haplotype region respectively; *r*^2^ is the square of the correlation coefficient.

The linkage disequilibrium analysis of the tested polymorphisms in TSHR and CTLA-4 revealed that four TSHR SNPs rs179247, rs2284722, rs12101261, and rs4903964, two SNPs (rs2300525 and rs17111394), and three SNPs in CTLA-4 (rs231804, rs1024161, and rs231726), respectively, formed three haplotype regions, each with a linkage disequilibrium coefficient ∣D′∣ > 0.7 (Figures 1 and 2 and Table 4; ∣D′∣).

Eight different haplotypes were identified based on the rs179247, rs2284722, rs12101261, and rs4903964 sites in the TSHR gene. The TSHR sites rs2300525 and rs17111394 composed three different haplotypes, while four different haplotypes were identified based on the rs231804, rs1024161, and rs231726 alleles of CTLA-4. The relationship between these haplotypes and GD revealed that haplotypes AGTA, GGCG, and AATA, haplotype CC, and haplotype AAA, respectively, were associated with the risk of GD (Table 5). No correlation was observed between TSHR and CTLA-4 genotypes and clinical phenotypes (Table 6).

Using MDR analysis of TSHR and CTLA-4 multiple site interaction yielded a significance of *P* > 0.05, indicating that there is no interaction between TSHR and CTLA-4 polymorphisms (Table 7). False-positive report probabilities (FPRPs) for the identified SNPs significantly associated with GD were <0.2 for rs179247, rs12101261, and rs4903964 (Table 8).

## 4. Discussion

GD is a common autoimmune thyroid disease. The prevalence of clinical hyperthyroidism in China is about 0.8%, and 80% of these cases are the result of GD. GD develops as a consequence of complex interactions between genetic, environmental, and immunological factors. TRAb is the most frequently encountered autoantibodies in those with GD (present in >90% of patients), and it can compete with TSH for TSHR binding. TSAb is a pathogenic antibody associated with GD, and recent work has shown that TSAb is linked with oxidative stress present in those with GD [28]. Oxidative stress is associated with GD inflammation. These TSHR agonist antibodies that trigger TSHR signaling are characteristic of GD. Genetic polymorphisms both in TSHR and in immune genes that regulate central and peripheral tolerance, such as CTLA-4 which constrains T cell activation, are linked to the pathogenesis of GD. As such, we assessed SNPs in both TSHR and CTLA-4 in the present study.

We confirmed that all 6 TSHR SNPS and all 4 CTLA-4 SNPs conformed to the Hardy-Weinberg equilibrium, indicating that our research subjects were a good representative population (*P* > 0.05).

We next analyzed the correlation of the six studied TSHR SNPs with GD, and through different analysis models, we identified the risk alleles and genotypes for GD associated with these sites.

For TSHR site rs179247, GD patients had a higher frequency of the A allele than healthy controls, and this allele was found to be the primary risk factor. In contrast, the G allele was negatively correlated with GD in the total population and in the individual male and female populations. Bufalo et al. demonstrated that TSHR intronic polymorphisms are associated with GD and Graves' ophthalmopathy susceptibility in a Brazilian population, with the AA genotype for rs179247 increasing GD risk [29]. rs179247 SNP AA or AG individuals have significantly lower TSHR mRNA expression levels in the thymus in nonautoimmune donors relative to those with the GG phenotype [30]. An analysis of polymorphisms in TSHR rs179247 pertaining to the pathogenesis of autoimmune thyroid diseases in children found that A alleles were more frequent in patients with GD in comparison to healthy subjects and that polymorphisms at this site contributed to the development of AITDs in children [21].

Several meta-analyses of this site have reported that it is associated with GD [31–33], and our study results are consistent with these reports.

In our study, the main GD risk factor at site rs2284722 was the A allele; A alleles were positively correlated with GD incidence in both the total population and the female population, and the AA genotype was associated with a higher risk for GD, especially in females.

For TSHR site rs12101261, the C alleles were negatively correlated with the incidence of GD in the total population, the male population, and the female population. The GD main risk factor for rs12101261 was the T alleles. There have been reports identifying this site as a possible causal SNP for GD susceptibility in the TSHR gene, potentially serving as a genetic marker to predict the outcome of persistent TSHR autoantibody positivity in GD patients [34]. Research has shown that the rs12101261 disease-associated T alleles are associated with lower TSHR expression in the thymus [16].

The main GD risk factors for rs4903964 were the A alleles; the G alleles were negatively correlated with the incidence of GD in the total population, the male population, and the female population.

There were no difference in the distribution of alleles or genotypes at rs2300525 between the case group and the control group (*P* > 0.05). We further analyzed the correlation of TSHR rs2300525 alleles with GD, and we found that the C allele was positively correlated with GD incidence only in the female population and that the risk of GD was 1.25x for C alleles relative to T alleles.

For TSHR site rs17111394, the C alleles were positively correlated with the incidence of GD in the total population and the female population. The risk of GD in mutant homozygous carriers was 1.78x and 2.15x, respectively, relative to homozygous TT carriers.

Linkage disequilibrium is a measure of the correlation between alleles at different loci. The linkage disequilibrium analysis of the four TSHR SNP sites rs179247, rs2284722, rs12101261, and rs4903964 and the linkage disequilibrium coefficient ∣D′∣ were all > 0.7, indicating that these four SNP sites were located in the same haplotype region. Two polymorphism sites, rs2300525 and rs17111394, have an imbalance coefficient of ∣D′∣ > 0.7, indicating that these two SNP sites are located in the same haplotype region.

The TSHR SNPs rs179247, rs2284722, rs12101261, and rs4903964 together formed 8 distinct haplotypes. We analyzed the relationship between these haplotypes and GD. Haplotypes AGTA, GGCG, and AATA were the most frequent of all haplotypes, accounting for more than 80 percent of the entire population. There was a positive correlation between GD risk and haplotypes AGTA and AATA. The risk was negatively correlated with the haplotypes GGCG and GACG; however, the GACG haplotype was a very small percentage of the study population. No association with GD was found for the other haplotypes (*P* > 0.05). For the rs2300525 and rs17111394 haplotypes, there was a positive correlation between haplotype CC and GD risk.

As for CTLA-4, there was no difference in the distribution of genotypes at four sites on the CTLA-4 gene between the case group and the control group (*P* > 0.05), while the distribution of alleles at rs231726 between the two groups was statistically significant (*P* < 0.05). CTLA-4 rs231726-G alleles were negatively correlated with the risk of GD in the total population, and the risk of GD was 0.73x for G allele carriers relative to A allele carriers. In the dominant model, the risk of GD in the total population was 0.78x for GG+AG carriers relative to homozygous AA carriers. In the dominant model of the rs10197319 site, TT+CT carriers had a 0.68x risk of GD in the total population relative to homozygous CC carriers, and the risk of GD in the male population was 0.32x relative to homozygous CC carriers. No correlation was found between the rs231804 and rs1024161 sites and GD risk. The linkage disequilibrium coefficients of three polymorphism sites, namely, rs231804, rs1024161, and rs231726, ∣D′∣ > 0.7, indicated that these 3 SNP sites were located in the same haplotype region.

We further analyzed the relationship between GD and the haplotypes formed by CTLA-4 SNP sites rs231804, rs1024161, and rs231726. A positive correlation was identified between haplotype AAA and GD risk (*P* < 0.05). No association with GD was found for the other haplotypes (*P* > 0.05).

We analyzed comparisons between TSHR and CTLA-4 genotypes and clinical GD characteristics such as diffuse goiter, exophthalmos, and different levels of TRAb, but we did not identify any correlations between these variables (*P* > 0.05). It is important to note that many patients were being treated using antithyroid drugs which have the potential to alter TRAb/TSH levels and to impact these clinical characteristics.

GD is a complex disease triggered by multiple genes and other factors. The information provided by a single SNP site is thus very limited in the study of such complex diseases. The variation and the interaction between multiple SNP sites are what ultimately lead to the development of such complex diseases. The pathogenesis of GD is related to the interaction between genes and the environment [35, 36]. We therefore analyzed the interactions between TSHR and CTLA-4 polymorphisms to assess their impact on GD. The best cross-validation consistency was for the single-factor model rs179247, whose training balance precision was 0.5864, the test set balance precision is 0.5864, and the cross-validation consistency rate is 10/10, but *P* > 0.05. Therefore, no interaction between TSHR gene and CTLA-4 gene was found.

We calculated the FPRP for the TSHR and CTLA-4 SNPS significantly associated with GD, yielding values < 0.2 for rs179247, rs12101261, and rs4903964, indicating that the association between these genotypes and GD was significant.

In conclusion, genetic factors play an important role in the development of GD. The five studied SNPs in TSHR intron 1 and the linkage disequilibrium haplotype composed of those related loci were all associated with GD. Only one linkage disequilibrium haplotype (AAA, composed of CTLA-4 sites rs231804, rs1024161, and rs231726) was found to be related to GD.

Previously, functional SNPs associated with diseases have been found to be more concentrated in the regulatory or coding regions of the genome. In contrast, most SNPs in the noncoding regions of the genome have been ignored by researchers. Most detected SNPs are normal variants and do not have biological functions. In spite of the fact that these SNPs function weakly or are even nonfunctional, their combinations often show a good correlation with disease or related phenotypes. To detect different specific SNP combinations associated with disease groups and to analyze the combination of these SNPs, it is possible to determine the indicators of disease sensitivity and predict therapeutic efficacy with the goal of improving early treatment and prevention. There have been many reports regarding the relationship between TSHR and CTLA-4 and GD in different populations [17, 19, 29, 37], including the Chinese Han population, revealing polymorphisms in these two genes to be related to GD. While these studies assessed different sites and populations, they all employed case-control methodology, as we did in the present study. We also had the advantage of assessing more SNPs than previous studies and of using different statistical models to achieve a more in-depth statistical analysis. Even so, our results were limited by the fact that our study included medicated patients, potentially impacting the association between identified genotypes and clinical presentation. While our results are promising, any statistical tests have the potential for false-positive results or correlations due to multiple confounding variables such as sample size or population stratification. Therefore, these results need to be further verified in a larger sample and among different populations.

## Figures and Tables

**Table 1:** Clinical data.

Basic information		Case group (*n* = 597)	Control group (*n* = 620)	*t*/*χ*^2^	*P*
Sex	Male	127	113	1.78	0.18
	Female	470	507		
Age		38.80 ± 14.20	44.29 ± 14.14	6.76	<0.001^∗^
Diffuse goiter	0	15	—	—	—
	I	186	—	—	—
	II	386	—	—	—
	III	10	—	—	—
Exophthalmos	Yes	252	—	—	—
	No	345	—	—	—
TRAb	≥1.5 U/L	456	—	—	—
	<1.5 U/L	96			

**Table 2:** Genotype distribution of TSHR and CTLA-4 gene and Hardy-Weinberg equilibrium test.

	SNPs	Case group (*n* = 597)	Control group (*n* = 620)	*χ* ^2^	*P*	HWE (*P*)
		*n* (%)	*n* (%)			
TSHR	**rs179247 (A>G)**					0.80
	A	895 (74.96)	782 (63.06)	40.16	<0.001^∗^	
	G	299 (25.04)	458 (36.94)			
	AA	342 (57.29)	248 (40.00)	39.44	<0.001^∗^	
	AG	211 (35.34)	286 (46.13)			
	GG	44 (7.37)	86 (13.87)			
	**rs2284722 (G>A)**					0.06
	G	833 (69.77)	937 (75.56)	10.31	0.001^∗^	
	A	361 (30.23)	303 (24.44)			
	GG	291 (48.74)	345 (55.65)	12.97	0.002^∗^	
	AG	251 (42.04)	247 (39.84)			
	AA	55 (9.22)	28 (4.51)			
	**rs12101261 (T>C)**					0.56
	T	872 (73.03)	756 (60.97)	39.97	<0.001^∗^	
	C	322 (26.97)	484 (39.03)			
	TT	320 (53.60)	234 (37.74)	38.60	<0.001^∗^	
	CT	232 (38.86)	288 (46.45)			
	CC	45 (7.54)	98 (15.81)			
	**rs4903964 (A>G)**					0.29
	A	824 (69.01)	701 (56.53)	40.49	<0.001^∗^	
	G	370 (30.99)	539 (43.47)			
	AA	279 (46.73)	205 (33.06)	41.47	<0.001^∗^	
	AG	266 (44.56)	291 (46.94)			
	GG	52 (8.71)	124 (20.00)			
	rs2300525 (T>C)					0.68
	T	836 (70.02)	909 (73.31)	3.24	0.072	
	C	358 (29.98)	331 (26.69)			
	TT	297 (49.75)	335 (54.03)	3.26	0.196	
	CT	242 (40.54)	239 (38.55)			
	CC	58 (9.71)	46 (7.42)			
	**rs17111394 (T>C)**					0.61
	T	913 (76.47)	1001 (80.73)	6.57	0.010^∗^	
	C	281 (23.53)	239 (19.27)			
	TT	355 (59.46)	406 (65.48)	6.55	0.038^∗^	
	CT	203 (34.00)	189 (30.48)			
	CC	39 (6.54)	25 (4.04)			
CTLA-4	rs231804 (A>G)					1.00
	A	1031 (86.35)	1036 (83.55)	3.72	0.054	
	G	163 (13.65)	204 (16.45)			
	AA	447 (74.87)	432 (69.68)	4.10	0.129	
	AG	137 (22.95)	172 (27.74)			
	GG	13 (2.18)	16 (2.58)			
	rs1024161 (A>G)					0.44
	A	876 (73.37)	868 (70.00)	3.40	0.065	
	G	318 (26.63)	372 (30.00)			
	AA	326 (54.61)	308 (49.68)	3.30	0.192	
	AG	224 (37.52)	252 (40.65)			
	GG	47 (7.87)	60 (9.67)			
	**rs231726 (A>G)**					0.79
	A	813 (68.09)	794 (64.03)	4.47	0.035^∗^	
	G	381 (31.91)	446 (35.97)			
	AA	283 (47.40)	256 (41.29)	4.75	0.093	
	AG	247 (41.37)	282 (45.48)			
	GG	67 (11.23)	82 (13.23)			
	rs10197319 (C>T)					0.45
	C	937 (78.48)	938 (75.65)	2.76	0.097	
	T	257 (21.52)	302 (24.35)			
	CC	367 (61.48)	351 (56.61)	3.00	0.223	
	CT	203 (34.00)	236 (38.07)			
	TT	27 (4.52)	33 (5.32)			

HWE: Hardy-Weinberg equilibrium; ^∗^*P* < 0.05.

**Table 3:** Multifactor logistic regression analyzes the correlation of TSHR and CTLA-4 SNPs with GD.

	SNPs		Alleles		Dominant model		Recessive model		Homozygous model		Heterozygous model	
			OR (95% CI)	*P*	OR (95% CI)	*P*	OR (95% CI)	*P*	OR (95% CI)	*P*	OR (95% CI)	*P*
TSHR	**rs179247**		G/A		GG+AG/AA		GG/AG+AA		GG/AA		AG/AA	
	TP		0.57 (0.48, 0.68)	<0.001^∗^	0.50 (0.40, 0.63)	<0.001^∗^	0.50 (0.34, 0.73)	<0.001^∗^	0.37 (0.25, 0.55)	<0.001^∗^	0.54 (0.42, 0.68)	<0.001^∗^
	Sex	M	0.56 (0.38, 0.84)	0.005^∗^	0.57 (0.34, 0.95)	0.030^∗^	0.30 (0.11, 0.80)	0.016^∗^	0.25 (0.09, 0.69)	0.007^∗^	0.67 (0.39, 1.15)	0.148
		F	0.57 (0.47, 0.70)	<0.001^∗^	0.48 (0.37, 0.62)	<0.001^∗^	0.55 (0.36, 0.83)	0.005^∗^	0.40 (0.26, 0.62)	<0.001^∗^	0.51 (0.39, 0.66)	<0.001^∗^
	**rs2284722**		A/G		AA+AG/GG		AA/AG+GG		AA/GG		AG/GG	
	TP		1.34 (1.12, 1.60)	0.001^∗^	1.33 (1.06, 1.67)	0.013^∗^	2.14 (1.34, 3.42)	0.002^∗^	2.33 (1.44, 3.78)	0.001^∗^	1.22 (0.96, 1.55)	0.097
	Sex	M	0.91 (0.61, 1.38)	0.669	0.90 (0.54, 1.51)	0.694	0.88 (0.34, 2.30)	0.797	0.85 (0.32, 2.28)	0.748	0.91 (0.53, 1.57)	0.744
		F	1.47 (1.21, 1.80)	<0.001^∗^	1.46 (1.14, 1.88)	0.003^∗^	2.79 (1.61, 4.83)	<0.001^∗^	3.15 (1.79, 5.53)	<0.001^∗^	1.31 (1.01, 1.70)	0.046^∗^
	**rs12101261**		C/T		CC+CT/TT		CC/CT+TT		CC/TT		CT/TT	
	TP		0.58 (0.49, 0.69)	<0.001^∗^	0.53 (0.42, 0.66)	<0.001^∗^	0.44 (0.30, 0.63)	<0.001^∗^	0.34 (0.23, 0.50)	<0.001^∗^	0.59 (0.46, 0.75)	<0.001^∗^
	Sex	M	0.58 (0.40, 0.86)	0.006^∗^	0.54 (0.32, 0.90)	0.018^∗^	0.43 (0.18, 1.01)	0.053	0.34 (0.14, 0.82)	0.017^∗^	0.60 (0.35, 1.04)	0.066
		F	0.58 (0.48, 0.70)	<0.001^∗^	0.52 (0.41, 0.68)	<0.001^∗^	0.44 (0.29, 0.66)	<0.001^∗^	0.34 (0.22, 0.52)	<0.001^∗^	0.59 (0.45, 0.77)	<0.001^∗^
	**rs4903964**		G/A		GG+AG/AA		GG/AG+AA		GG/AA		AG/AA	
	TP		0.58 (0.50, 0.69)	<0.001^∗^	0.57 (0.45, 0.71)	<0.001^∗^	0.38 (0.27, 0.54)	<0.001^∗^	0.31 (0.21, 0.45)	<0.001^∗^	0.67 (0.53, 0.86)	0.020^∗^
	Sex	M	0.60 (0.41, 0.87)	0.008^∗^	0.56 (0.33, 0.94)	0.027^∗^	0.46 (0.21, 0.98)	0.044^∗^	0.36 (0.16, 0.82)	0.014^∗^	0.64 (0.37, 1.10)	0.107
		F	0.58 (0.48, 0.70)	<0.001^∗^	0.57 (0.44, 0.74)	<0.001^∗^	0.37 (0.25, 0.54)	<0.001^∗^	0.30 (0.20, 0.45)	<0.001^∗^	0.68 (0.52, 0.90)	0.007^∗^
	rs2300525		C/T		CC+CT/TT		CC/CT+TT		CC/TT		CT/TT	
	TP		1.18 (0.99, 1.40)	0.072	1.18 (0.95, 1.48)	0.143	1.34 (0.90, 2.01)	0.154	1.42 (0.93, 2.15)	0.101	1.14 (0.90, 1.44)	0.288
	Sex	M	0.92 (0.62, 1.36)	0.668	0.87 (0.53, 1.45)	0.600	0.98 (0.40, 2.39)	0.959	0.91 (0.36, 2.32)	0.849	0.87 (0.51, 1.47)	0.593
		F	1.25 (1.03, 1.52)	0.028^∗^	1.28 (0.99, 1.64)	0.058	1.45 (0.92, 2.29)	0.106	1.58 (0.99, 2.52)	0.055	1.22 (0.93, 1.59)	0.146
	**rs17111394**		C/T		CC+CT/TT		CC/CT+TT		CC/TT		CT/TT	
	TP		1.29 (1.06, 1.57)	0.010^∗^	1.29 (1.02, 1.63)	0.031^∗^	1.66 (0.99, 2.78)	0.055	1.78 (1.05, 3.00)	0.031^∗^	1.22 (0.96, 1.56)	0.111
	Sex	M	1.02 (0.66, 1.57)	0.934	1.06 (0.63, 1.78)	0.834	0.88 (0.30, 2.60)	0.822	0.91 (0.30, 2.72)	0.865	1.06 (0.61, 1.82)	0.844
		F	1.37 (1.10, 1.70)	0.005^∗^	1.36 (1.05, 1.76)	0.021^∗^	1.99 (1.10, 3.59)	0.023^∗^	2.15 (1.18, 3.91)	0.012^∗^	1.26 (0.96, 1.66)	0.092
CTLA-4	rs231804		G/A		GG+AG/AA		GG/AG+AA		GG/AA		AG/AA	
	TP		0.80 (0.64, 1.00)	0.054	0.77 (0.60, 1.00)	0.045	0.86 (0.41, 1.80)	0.680	0.80 (0.38, 1.86)	0.556	0.77 (0.59, 1.00)	0.050
	Sex	M	0.66 (0.39, 1.11)	0.116	0.69 (0.39, 1.23)	0.203	—	—	—	—	0.75 (0.42, 1.36)	0.346
		F	0.84 (0.66, 1.08)	0.176	0.80 (0.60, 1.05)	0.108	1.08 (0.50, 2.36)	0.845	1.01 (0.46, 2.21)	0.977	0.78 (0.58, 1.04)	0.086
	rs1024161		G/A		GG+AG/AA		GG/AG+AA		GG/AA		AG/AA	
	TP		0.85 (0.71, 1.01)	0.065	0.82 (0.66, 1.03)	0.085	0.81 (0.54, 1.20)	0.293	0.75 (0.49, 1.13)	0.162	0.84 (0.66, 1.06)	0.145
	Sex	M	0.93 (0.62, 1.39)	0.712	0.94 (0.57, 1.57)	0.825	0.77 (0.27, 2.18)	0.617	0.76 (0.26, 2.22)	0.612	0.98 (0.58, 1.65)	0.929
		F	0.83 (0.68, 1.01)	0.065	0.79 (0.62, 1.02)	0.070	0.81 (0.53, 1.26)	0.351	0.74 (0.48, 1.16)	0.192	0.81 (0.62, 1.05)	0.112
	rs231726		G/A		GG+AG/AA		GG/AG+AA		GG/AA		AG/AA	
	TP		**0.83 (0.71, 0.99)**	**0.035** ^∗^	**0.78 (0.62, 0.98)**	**0.031** ^∗^	0.83 (0.59, 1.18)	0.300	0.74 (0.52, 1.07)	0.108	0.79 (0.62, 1.01)	0.055
	Sex	M	0.76 (0.52, 1.12)	0.163	0.74 (0.44, 1.23)	0.248	0.62 (0.27, 1.41)	0.254	0.55 (0.23, 1.31)	0.177	0.79 (0.46, 1.36)	0.398
		F	0.85 (0.71, 1.03)	0.097	0.79 (0.61, 1.02)	0.067	0.89 (0.61, 1.30)	0.541	0.79 (0.53, 1.18)	0.252	0.79 (0.60, 1.03)	0.084
	rs10197319		T/C		TT+CT/CC		TT/CT+CC		TT/CC		CT/CC	
	TP		0.85 (0.70, 1.03)	0.097	**0.68 (0.54, 0.86)**	**0.001** ^∗^	0.85 (0.51, 1.43)	0.543	0.75 (0.44, 1.28)	0.291	**0.67 (0.53, 0.86)**	**0.001** ^∗^
	Sex	M	0.94 (0.62, 1.45)	0.793	**0.32 (0.18, 0.57)**	**<0.001** ^∗^	1.12 (0.29, 4.27)	0.872	0.78 (0.20, 3.03)	0.725	**0.28 (0.15, 0.52)**	**<0.001** ^∗^
		F	0.83 (0.67, 1.03)	0.085	0.80 (0.62, 1.03)	0.080	0.81 (0.46, 1.43)	0.466	0.75 (0.42, 1.33)	0.319	0.80 (0.62, 1.05)	0.107

TP: total population; M: male; F: female; ^∗^*P* < 0.05.

**Table 4:** Linkage disequilibrium analysis of 4 loci and 2 loci of the TSHR gene and 3 loci of the CTLA-4 gene.

∣D′∣					
		rs179247	rs2284722	rs12101261	rs4903964
*r* ^2^	rs179247	—	*0.845*	*0.955*	*0.862*
	rs2284722	0.121	—	*0.85*	*0.793*
	rs12101261	0.832	0.134	—	*0.881*
	rs4903964	0.562	0.141	0.645	—
		rs2300525	rs17111394		
	rs2300525	—	*0.977*		
	rs17111394	0.657	—		
		rs231804	rs1024161	rs231726	
	rs231804	—	*0.987*	*0.99*	
	rs1024161	0.437	—	*0.993*	
	rs231726	0.338	0.758	—	

The italic part represents ∣D′∣, and the non-italic part represents *r*^2^.

**Table 5:** The relationship between haplotypes and GD.

SNP	Haplotype	Case group		Control group		OR (95% CI)	*P*
		*n* = 597	%	*n* = 620	%		
rs179247	**AGTA**	224	37.6	200	32.2	1.27 (1.07, 1.50)	0.005^∗^
rs2284722	**GGCG**	127	21.3	203	32.7	0.56 (0.46, 0.67)	<0.001^∗^
rs12101261	**AATA**	168	28.2	132	21.3	1.45 (1.21, 1.75)	<0.001^∗^
rs4903964	AGTG	32	5.3	33	5.3	1.00 (0.70, 1.43)	0.997
	AGCG	14	2.4	16	2.6	0.94 (0.57, 1.57)	0.820
	GGCA	11	1.9	10	1.6	1.20 (0.66, 2.20)	0.548
	AATG	6	1.0	8	1.3	0.76 (0.36, 1.64)	0.488
	GACG	4	0.6	9	1.5	0.40 (0.18, 0.92)	0.031^∗^
rs2300525	TT	417	69.8	451	72.8	0.86 (0.73, 1.03)	0.105
rs17111394	**CC**	139	23.3	117	18.8	1.32 (1.08, 1.60)	0.006^∗^
	CT	39	6.6	49	7.9	0.83 (0.61, 1.12)	0.223
rs231804	**AAA**	405	67.9	395	63.7	1.21 (1.02, 1.43)	0.029^∗^
rs1024161	GGG	81	13.5	102	16.4	0.80 (0.64, 1.00)	0.046
rs231726	AGG	79	13.2	83	13.4	0.98 (0.78, 1.24)	0.863
	AAG	32	5.3	38	6.2	0.84 (0.60, 1.19)	0.323

Four of the eight haplotypes (composed of rs179247, rs2284722, rs12101261, and rs4903964) had frequencies greater than 0.03. There are four haplotypes (composed of rs231804, rs1024161, and rs231726) that had frequencies greater than 0.03. ^∗^*P* < 0.05.

**Table 6:** Correlation between TSHR and CTLA-4 SNPs and clinical phenotypes.

			Goiter						Exophthalmos				TRAb (U/L)			
	SNP	Genotype	0	I	II	III	*χ* ^2^	*P*	Yes	No	*χ* ^2^	*P*	≥1.5	<1.5	*χ* ^2^	*P*
TSHR	rs179247	AA	7	99	232	4	3.67	0.30	150	192	1.11	0.57	274	47	4.23	0.12
		AG	7	71	127	6			83	128			153	40		
		GG	1	16	27	0			19	25			29	9		
	rs2284722	GG	9	81	197	4	1.10	0.78	119	172	0.41	0.82	215	53	2.72	0.26
		AG	6	87	153	5			109	142			194	37		
		AA	0	18	36	55			24	31			47	6		
	rs12101261	TT	7	103	204	6	3.14	0.37	136	184	0.17	0.92	260	43	4.84	0.09
		CT	6	68	154	4			96	136			164	45		
		CC	2	15	28	0			20	25			32	8		
	rs4903964	AA	7	86	179	7	2.61	0.46	121	158	0.32	0.85	226	36	5.60	0.06
		AG	5	83	175	3			109	157			192	53		
		GG	3	17	32	0			22	30			38	7		
	rs2300525	TT	8	90	195	4	3.48	0.32	129	168	1.62	0.45	227	49	0.11	0.95
		CT	5	76	155	6			103	139			184	37		
		CC	2	20	36	0			20	38			45	10		
	rs17111394	TT	10	111	230	4	2.87	0.41	153	202	2.24	0.33	267	63	4.42	0.11
		CT	4	58	135	6			87	116			161	24		
		CC	1	17	21	0			12	27			28	9		
CTLA-4	rs231804	AA	12	138	288	9	1.54	0.67	187	260	1.03	0.60	345	72	0.50	0.78
		AG	3	45	88	1			61	76			102	21		
		GG	0	3	10	0			4	9			9	3		
	rs1024161	AA	11	101	207	7	2.56	0.46	140	186	0.78	0.68	250	53	1.51	0.47
		AG	2	69	150	3			95	129			174	33		
		GG	2	16	29	0			17	30			32	10		
	rs231726	AA	9	86	184	4	0.70	0.87	123	160	0.84	0.66	218	46	0.84	0.66
		AG	4	81	157	5			104	143			190	37		
		GG	2	19	45	1			25	42			48	13		
	rs10197319	CC	12	108	239	8	4.78	0.19	157	210	0.71	0.70	282	58	0.14	0.93
		CT	3	69	129	2			82	121			153	34		
		TT	0	9	18	0			13	14			21	4		

**Table 7:** MDR analysis of TSHR and CTLA-4 multiple site interaction model.

Model	Training set balance precision	Test set balance precision	Cross-validation consistency	*P*	OR (95% CI)
rs179247	0.5864	0.5864	10/10	0.0565	2.01 (0.98, 4.14)
rs179247, rs4903964	0.5919	0.5734	6/10	0.1053	1.81 (0.88, 3.71)
rs179247, rs4903964, rs10197319	0.6074	0.5706	5/10	0.1193	1.77 (0.86, 3.62)

**Table 8:** False-positive report probability.

SNP	OR (95% CI)	Prior probability			
		0.25	0.1	0.01	0.001
rs179247	0.57 (0.48, 0.68)	<0.001	<0.001	<0.001	<0.001
rs2284722	1.34 (1.12, 1.60)	0.004	0.012	0.119	0.576
rs12101261	0.58 (0.49, 0.69)	<0.001	<0.001	<0.001	<0.001
rs4903964	0.58 (0.50, 0.69)	<0.001	<0.001	<0.001	<0.001
rs17111394	1.29 (1.06, 1.57)	0.034	0.096	0.540	0.922
rs231726	0.83 (0.71, 0.99)	0.104	0.258	0.792	0.975

Preset the critical value = 0.2; OR = 1.5.

## Data Availability

The genotype data used to support the findings of this study are restricted by the Medical Ethics Committee of The First Affiliated Hospital of Bengbu Medical College in order to protect patient privacy. Data are available from the author sunwh2007@163.com for researchers who meet the criteria for access to confidential data.

## References

[1] Shukla S. K., Singh G., Ahmad S., Pant P. (2018). Infections, genetic and environmental factors in pathogenesis of autoimmune thyroid diseases. *Microbial Pathogenesis*.

[2] Dittmar M., Libich C., Brenzel T., Kahaly G. J. (2011). Increased familial clustering of autoimmune thyroid diseases. *Hormone and Metabolic Research*.

[3] Hemminki K., Li X., Sundquist J., Sundquist K. (2010). The epidemiology of Graves disease: evidence of a genetic and an environmental contribution. *Journal of Autoimmunity*.

[4] McLeod D. S. A., Cooper D. S., Ladenson P. W., Whiteman D. C., Jordan S. J. (2015). Race/ethnicity and the prevalence of thyrotoxicosis in young Americans. *Thyroid*.

[5] Chu X., Pan C. M., Zhao S. X. (2011). A genome-wide association study identifies two new risk loci for Graves' disease. *Nature Genetics*.

[6] Cooper J. D., Simmonds M. J., Walker N. M. (2012). Seven newly identified loci for autoimmune thyroid disease. *Human Molecular Genetics*.

[7] Eriksson N., Tung J. Y., Kiefer A. K. (2012). Novel associations for hypothyroidism include known autoimmune risk loci. *PLoS One*.

[8] Zhao S. X., Xue L. Q., Liu W. (2013). Robust evidence for five new Graves disease risk loci from a staged genome-wide association analysis. *Human Molecular Genetics*.

[9] Chu X., Shen M., Xie F. (2013). An X chromosome-wide association analysis identifies variants in *GPR174* as a risk factor for Graves disease. *Journal of Medical Genetics*.

[10] Kwak S. H., Park Y. J., Go M. J. (2014). A genome-wide association study on thyroid function and anti-thyroid peroxidase antibodies in Koreans. *Human Molecular Genetics*.

[11] Medici M., Porcu E., Pistis G. (2014). Identification of novel genetic loci associated with thyroid peroxidase antibodies and clinical thyroid disease. *PLoS Genetics*.

[12] Oryoji D., Ueda S., Yamamoto K. (2015). Identification of a Hashimoto thyroiditis susceptibility locus via a genome-wide comparison with Graves disease. *The Journal of Clinical Endocrinology and Metabolism*.

[13] Brčić L., Barić A., Gračan S. (2019). Genome-wide association analysis suggests novel loci for Hashimoto’s thyroiditis. *Journal of Endocrinological Investigation*.

[14] Zhan M., Chen G., Pan C. M. (2014). Genome-wide association study identifies a novel susceptibility gene for serum TSH levels in Chinese populations. *Human Molecular Genetics*.

[15] Jansson L., Vrolix K., Jahraus A., Martin K. F., Wraith D. C. (2018). Immunotherapy with apitopes blocks the immune response to TSH receptor in HLA-DR transgenic mice. *Endocrinology*.

[16] Stefan M., Wei C., Lombardi A. (2014). Genetic-epigenetic dysregulation of thymic TSH receptor gene expression triggers thyroid autoimmunity. *Proceedings of the National Academy of Sciences of the United States of America*.

[17] Zhao S. X., Pan C. M., Cao H. M. (2010). Association of the CTLA-4 gene with Graves disease in the Chinese Han population. *PLoS One*.

[18] Ting W. H., Chien M. N., Lo F. S. (2016). Association of cytotoxic T-lymphocyte-associated protein 4 (CTLA-4) gene polymorphisms with autoimmune thyroid disease in children and adults: case-control study. *PLoS One*.

[19] Chen X., Hu Z., Liu M. (2018). Correlation between CTLA-4 and CD40 gene polymorphisms and their interaction in Graves disease in a Chinese Han population. *BMC Medical Genetics*.

[20] Ramgopal S., Rathika C., Padma Malini R., Murali V., Arun K., Balakrishnan K. (2019). Critical amino acid variations in HLA-DQB1^∗^ molecules confers susceptibility to autoimmune thyroid disease in South India. *Genes & Immunity*.

[21] Rydzewska M., Góralczyk A., Gościk J. (2018). Analysis of chosen polymorphisms rs2476601 a/G–PTPN22, rs1990760 C/T–IFIH1, rs179247 a/G–TSHR in pathogenesis of autoimmune thyroid diseases in children. *Autoimmunity*.

[22] Shehjar F., Afroze D., Misgar R. A., Malik S. A., Laway B. A. (2018). Association of FoxP3 promoter polymorphisms with the risk of Graves' disease in ethnic Kashmiri population. *Gene*.

[23] Li L., Ding X., Wang X. (2018). Polymorphisms of IKZF3 gene and autoimmune thyroid diseases: associated with Graves disease but not with Hashimoto’s thyroiditis. *Cellular Physiology and Biochemistry*.

[24] Wang D., Chen J., Zhang H., Zhang F., Yang L., Mou Y. (2017). Role of different CD40 polymorphisms in Graves disease and Hashimoto’s thyroiditis. *Immunological Investigations*.

[25] Khong J. J., Burdon K. P., Lu Y. (2016). Pooled genome wide association detects association upstream of FCRL3 with Graves’ disease. *BMC Genomics*.

[26] Lombardi A., Menconi F., Greenberg D. (2016). Dissecting the genetic susceptibility to Graves’ disease in a cohort of patients of Italian origin. *Frontiers in Endocrinology*.

[27] Gu L. Q., Zhu W., Zhao S. X. (2010). Clinical associations of the genetic variants of CTLA-4, Tg, TSHR, PTPN22, PTPN12 and FCRL3 in patients with Graves’ disease. *Clinical Endocrinology*.

[28] Diana T., Daiber A., Oelze M. (2018). Stimulatory TSH-receptor antibodies and oxidative stress in Graves disease. *The Journal of Clinical Endocrinology & Metabolism*.

[29] Bufalo N. E., dos Santos R. B., Marcello M. A. (2015). *TSHR* intronic polymorphisms (rs179247 and rs12885526) and their role in the susceptibility of the Brazilian population to Graves’ disease and Graves’ ophthalmopathy. *Journal of Endocrinological Investigation*.

[30] Colobran R., Armengol M. . P., Faner R. (2011). Association of an SNP with intrathymic transcription of *TSHR* and Graves' disease: a role for defective thymic tolerance. *Human Molecular Genetics*.

[31] Xiong H., Wu M., Yi H. (2016). Genetic associations of the thyroid stimulating hormone receptor gene with Graves diseases and Graves ophthalmopathy: a meta-analysis. *Scientific Reports*.

[32] Qian W., Xu K., Jia W. (2016). Association between TSHR gene polymorphism and the risk of Graves' disease: a meta-analysis. *Journal of Biomedical Research*.

[33] Gong J., Jiang S. J., Wang D. K. (2016). Association of polymorphisms of rs179247 and rs12101255 in thyroid stimulating hormone receptor intron 1 with an increased risk of Graves disease: a meta-analysis. *Journal of Huazhong University of Science and Technology. Medical Sciences*.

[34] Liu B. L., Yang S. Y., Liu W. (2014). Refined association of TSH receptor susceptibility locus to Graves disease in the Chinese Han population. *European Journal of Endocrinology*.

[35] Simmonds M. J. (2013). GWAS in autoimmune thyroid disease: redefining our understanding of pathogenesis. *Nature Reviews Endocrinology*.

[36] Dong Y. H., Fu D. G. (2014). Autoimmune thyroid disease: mechanism, genetics and current knowledge. *European Review for Medical and Pharmacological Sciences*.

[37] Liu L., Wu H.-Q., Wang Q. (2012). Association between thyroid stimulating hormone receptor gene intron polymorphisms and autoimmune thyroid disease in a Chinese Han population. *Endocrine Journal*.

